# Mapping the future: identifying research priorities in rheumatoid arthritis with the James Lind Alliance approach

**DOI:** 10.1186/s41927-025-00588-7

**Published:** 2025-11-12

**Authors:** Kristine Røren Nordén, Anna Fryxelius, Ingrid Fjeldheim Bånerud, Gunnstein Bakland, Tore Voksø, Mona Larsen, Astrid Torgersen Lunestad, Kjersti Storheim, Amy Martinsen, Rikke Munk Killingmo

**Affiliations:** 1 Health Services Research and Innovation Unit, Center for Treatment of Rheumatic and Musculoskeletal Diseases (REMEDY), Diakonhjemmet Hospital, Postboks 23 Vinderen, Oslo, 0319 Norway; 2 Norwegian Rheumatism Association, Oslo, Norway; 3 Department of Research and Innovation, Division of Clinical Neuroscience, Oslo University Hospital, Oslo, Norway; 4 Department of Rheumatology, University Hospital of North Norway, Tromsø, Norway; 5 Institute of Clinical Medicine, Faculty of Health, The Arctic University of Norway, Tromsø, Norway; 6 Norwegian Association for Pregnancy-related Pelvic Girdle Health (LKB), Oslo, Norway; 7 Department of Rehabilitation Science and Health Technology, Oslo Metropolitan University, Oslo, Norway

**Keywords:** Rheumatoid arthritis, Patient participation, Research strategy

## Abstract

**Background:**

Considerable knowledge gaps remain regarding the cause, prevention, and management of rheumatoid arthritis (RA), with limited systematic effort to establish research priorities that truly align with the preferences of those impacted by RA.

**Objectives:**

To identify and prioritize unanswered questions about RA, capturing insights from people living with RA and healthcare professionals involved it its management.

**Methods:**

A James Lind Alliance (JLA) Priority Setting Partnership was established and followed six steps: (1) form a steering group, (2) define scope, (3) collect evidence uncertainties using focus groups, (4) collate evidence uncertainties and verify by checking existing research, (5) shortlist evidence uncertainties in an online survey, and (6) identify the top 10 research priorities through a priority-setting workshop.

**Results:**

A total of 212 questions were generated from three focus group interviews and distilled into 36 questions for a survey distributed to people with RA and healthcare professionals. Among 554 responses (mean age 58 [SD 13] years; 481 [87%] women), 449 (81%) identified as people with RA, and 105 (19%) as healthcare professionals, with some reporting dual roles. The ranking process resulted in a shortlist of 26 questions, which were further narrowed down to the top 10 research priorities. The top 10 research priorities addressed strategies for RA prevention, rapid diagnosis, identifying effective treatments, reducing treatment side effects, and holistic management approaches.

**Conclusion:**

Adopting the JLA method, this study mapped out core research priorities in RA, offering valuable insights that can help researchers, policymakers, and funders align future RA research with patient and clinical needs.

**Clinical Trial number:**

Not applicable.

**Supplementary Information:**

The online version contains supplementary material available at 10.1186/s41927-025-00588-7.

## Introduction

Current rheumatological practice emphasizes a treat-to-target strategy, aiming for remission or low disease activity as the optimal outcomes for patients [1]. Despite these advances, gaps remain in our understanding of the cause of rheumatoid arthritis (RA), disease course, and treatment response, and many patients experience residual symptoms, highlighting the need for continued research [1–3]. 

Traditionally, researchers and funding bodies have predominantly dictated which research projects are pursued. However, in recent years, there has been an uptick in the involvement of public stakeholders in setting research priorities [4, 5]. This shift aims to ensure that future research aligns more closely with the needs and values of end-users, particularly in healthcare, where clinicians and patients can play a vital role in mapping research priorities [6]. The literature underscores the potential benefits of patient research partner involvement at various phases of rheumatology research, particularly in ensuring that the research agenda is relevant to people living with RA [7]. This focus aligns with the European Alliance of Associations for Rheumatology (EULAR) recommendations, emphasizing that patient research partners should have a seat at the table in shaping the research agenda [8]. Nonetheless, challenges such as power imbalances and feelings of being unheard persist, highlighting the need for supportive environments to foster collaboration and inclusivity [7]. 

One approach to ensure the research agenda addresses the needs of individuals impacted by a disease is the James Lind Alliance (JLA) method [9]. This method brings people living with the condition and clinicians together in Priority Setting Partnerships (PSPs) to jointly identify and prioritize unanswered questions and evidence uncertainties related to a specific condition. Key phases of JLA PSPs include establishing a steering committee, gathering evidence uncertainties from people living with the condition and clinicians, summarizing these uncertainties, conducting an interim prioritization, and holding a final workshop to agree on the top 10 research priorities [9]. While PSPs guided by the JLA method have been implemented across various conditions [5, 10]their application within rheumatology has been limited. The aim of the present study was therefore to identify and prioritize the top 10 research priorities for RA through a JLA PSP involving people living with the disease and healthcare professionals.

## Methods

This project, initiated by the Council for Musculoskeletal Health and the Norwegian Rheumatism Association, took place in Norway as part of a larger initiative aimed at enhancing patient involvement in research related to rheumatic and musculoskeletal conditions [11]. The target audience of the research priorities include researchers, policy makers, and funding organizations, with the primary beneficiaries being people living with RA. The PSP was performed in alignment with the JLA methodology, [9] and current study reporting was guided by the Reporting guideline for priority setting of health research (REPRISE) [12]. 

### Ethics approval and consent to participate

As outlined in the JLA guidebook, PSPs primarily involve service consultations with relevant stakeholders and typically fall outside the scope of health research authority approvals. Consequently, ethical approval is seldom required for PSPs [9]. The data protection officer at Oslo University Hospital, Oslo, Norway, was consulted and waived the need for ethical approval and consent to participate, as no identifiable data was collected or recorded throughout the PSP. Additionally, the data does not qualify as research data requiring ethical committee approval in Norway [11, 13]. Participants were fully informed about their roles as stakeholders in the study and the nature of their involvement, with assurance that all information and responses collected through focus group interviews and the survey (as detailed below) would be completely anonymized. All data were stored on a secure IT-platform at Oslo University Hospital, with restricted access to ensure confidentiality.

### Establishing a steering committee and defining scope

A steering committee was formed, inviting people living with RA through the Norwegian Rheumatism Association, alongside clinicians through the Norwegian Society for Rheumatology and the Norwegian Interdisciplinary Organization in Rheumatology. Clinical experience was essential for the healthcare professionals invited to the committee, although concurrent research experience was preferred to enhance the methodological rigor of the project. The resulting committee comprised three experienced patient research partners, two clinicians with research backgrounds (one consultant rheumatologist and one physiotherapist), a project leader (RMK) with both clinical and research expertise, and a deputy project leader (ATL) knowledgeable in patient research involvement and PSPs. A project protocol was developed, and the scope of the PSP was intentionally broad, reflecting the gap in PSPs for RA. The project scope was defined as ‘*What should be the focus of research to preserve or enhance the quality of life, improve function, reduce pain, and increase longevity for people with RA?’*.

### Identifying and collating evidence uncertainties

Focus group interviews were organized to gather evidence uncertainties related to the scope (outline of focus groups available in supplementary file 1). These sessions were facilitated and moderated by the project leader (RMK) and deputy project leader (ATL). Eligible participants included people living with RA and healthcare professionals involved in its clinical management. Two focus group interviews were held with people with RA; one was an in-person meeting and the other took place digitally on the Zoom platform to facilitate recruitment from rural areas of Norway. Additionally, one digital focus group interview was conducted with healthcare professionals. All focus group interviews lasted two hours. To acknowledge their contribution, focus group participants received a universal gift card valued at approximately €45. No data on age or RA disease duration (if applicable) was collected for focus group participants, in accordance with our intention to avoid gathering any identifiable data.

Following the focus groups, the project leader (RMK) sorted all proposed evidence uncertainties as either within or outside the project’s scope. Those identified as within scope were refined, duplicates were merged, and the evidence uncertainties were framed as questions and thematically organized to form a set of core research questions. The goal was to ensure that these questions were written in lay language and broadly framed, avoiding excessive specificity. This approach allowed for both open-ended and close-ended questions, accommodating a range of research designs to address the identified uncertainties. The steering committee reviewed the suggested core questions to ensure consensus on the consolidation of evidence uncertainties, ensuring that none were overlooked.

The core research questions were subsequently checked by the project leader (RMK) against current literature to determine whether they had been adequately addressed in previous studies. PubMed served as the search database and priority was given to high quality evidence, preferably systematic reviews and current RA treatment recommendations. In cases of uncertainty, the questions were brought before the steering committee for further evaluation. A question was retained unless there was substantial evidence indicating that it had been thoroughly investigated in prior aggregated research. All research questions that remained unanswered were included in the interim prioritization survey.

### Interim prioritization

An online survey (nettskjema.no) was developed to rate the core research questions and produce a shortlist for the final priority-setting workshop. The core research questions were thematically categorized under five theme categories: (1) Causes, risk factors, prevention, and prognosis, (2) Examination and diagnosis, (3) Treatment and side effects, (4) Recognition and attitudes, and (5) Societal measures and structure of the healthcare system. Respondents were asked to evaluate each core research question on a five-point scale: (1) Unimportant, (2) Neutral, (3) Slightly important, (4) Quite important, and (5) Very important. Given that all survey items emerged from focus groups to reflect important research topics, and the potential for polarized responses to survey items [14] we anticipated that the overall ratings of questions might skew towards higher values and complicate the selection of the top research questions. To address this, participants were also asked to rate the thematic categories using the same five-point scale, allowing us to account for the possibility that some categories might be weighted higher than others. Additionally, demographic information regarding age, gender, RA disease duration (if applicable), healthcare profession, and self-reported level of RA competence (if applicable) were collected. The survey was open for one month and circulated to a diverse audience of people with RA and clinicians across Norway through various channels, including the Norwegian Rheumatism Association, the Norwegian Society for Rheumatology, the Norwegian Interdisciplinary Organization in Rheumatology, social media platforms, and the networks of the steering committee.

Raw data from the survey were analysed to determine the core research questions that received the highest overall importance scores. The goal was to present the top 20 research questions, ranked both with and without adjustment for theme category ratings, at the final priority-setting workshop.

### Priority-setting workshop

The final priority setting workshop utilized the nominal group technique to reach consensus on the top 10 research priorities in RA. Invitations to the priority-setting workshop were sent out one month in advance, and participants received an agenda with the list of core questions one week prior to the workshop to help prepare for the discussion. Participants included both people living with RA and healthcare professionals. To maintain the integrity of our approach to avoid collecting identifiable data, no information was gathered on age or RA disease duration (if applicable). The participants were organized into two groups, each comprising both people living with RA and healthcare professionals.

Participants were handed a series of laminated cards with the core questions printed on the front. Mean scores from the interim prioritization questionnaire, categorized by scores from both people living with RA and healthcare professionals, were printed on the back of each card. The workshop was chaired by the project leader (RKM) and deputy project leader (ATL), who emphasized equal participation to ensure that everyone had the opportunity to express their views [9]. Workshop participants received a universal gift card (approximately €45) akin to the one awarded to focus group participants. Table 1 outlines the five phases of the priority-setting workshop.

**Table 1:** Five phases of the priority-setting workshop

Phase	Content
1) Small group discussion	Cards with core research questions on one side and rankings from interim prioritization survey on the other. Asked to identify top three important research questions and bottom three least important ones
2) First round of small group ranking	Organized the core research questions into categories; most important (gold), uncertain (silver), least important (bronze), and then prioritized all core questions in ranked order.
3) Collective group review	Rankings from the small groups compiled into a spreadsheet with each question assigned a rank. Group rankings presented to all participants in a plenary session, open to discussion
4) Second round of small group ranking	Same small groups reconvened to discuss and adjust ranked list based on the plenary discussion. Rearranged cards to reflect the new ranking order.
5) Final whole group review	Revised rankings from small groups assimilated in spreadsheet and presented in plenary. Final ranking discussed until consensus was reached among participants.

### Statistical analysis

Demographic information from survey respondents are presented with summary statistics as appropriate, with mean (SD) for continuous data, and counts and percentages for categorial data. The ratings for the core research questions in the survey were generally high, and it was difficult to rank them using median scores. As a result, mean (SD) scores were calculated, both for all respondents and separately for people living with RA and healthcare professionals. The overall mean scores were then used to rank the top core research questions, both with and without adjustment for thematic category weighting. The rankings that were adjusted for theme category ratings were computed by multiplying the overall mean rating of each core question by the overall mean rating of its respective theme category. The proportion of missing data was well below 5%, and no imputation was performed for missing values. All analyses were conducted using Excel (365, Microsoft, Redmond, USA) and STATA (v. 19, StataCorp, Texas, USA).

### Patient and public involvement

Three patient research partners (AF, TV, ML) and two clinicians (KRN and GB) seasoned in the management of RA served as members of the steering committee and had key roles in the PSP. They were actively involved in recruiting participants for the focus groups and the priority-setting workshop, as well as in developing strategies for disseminating the online survey. Additionally, they are co-authors of the current paper.

## Results

**Fig. 1:**
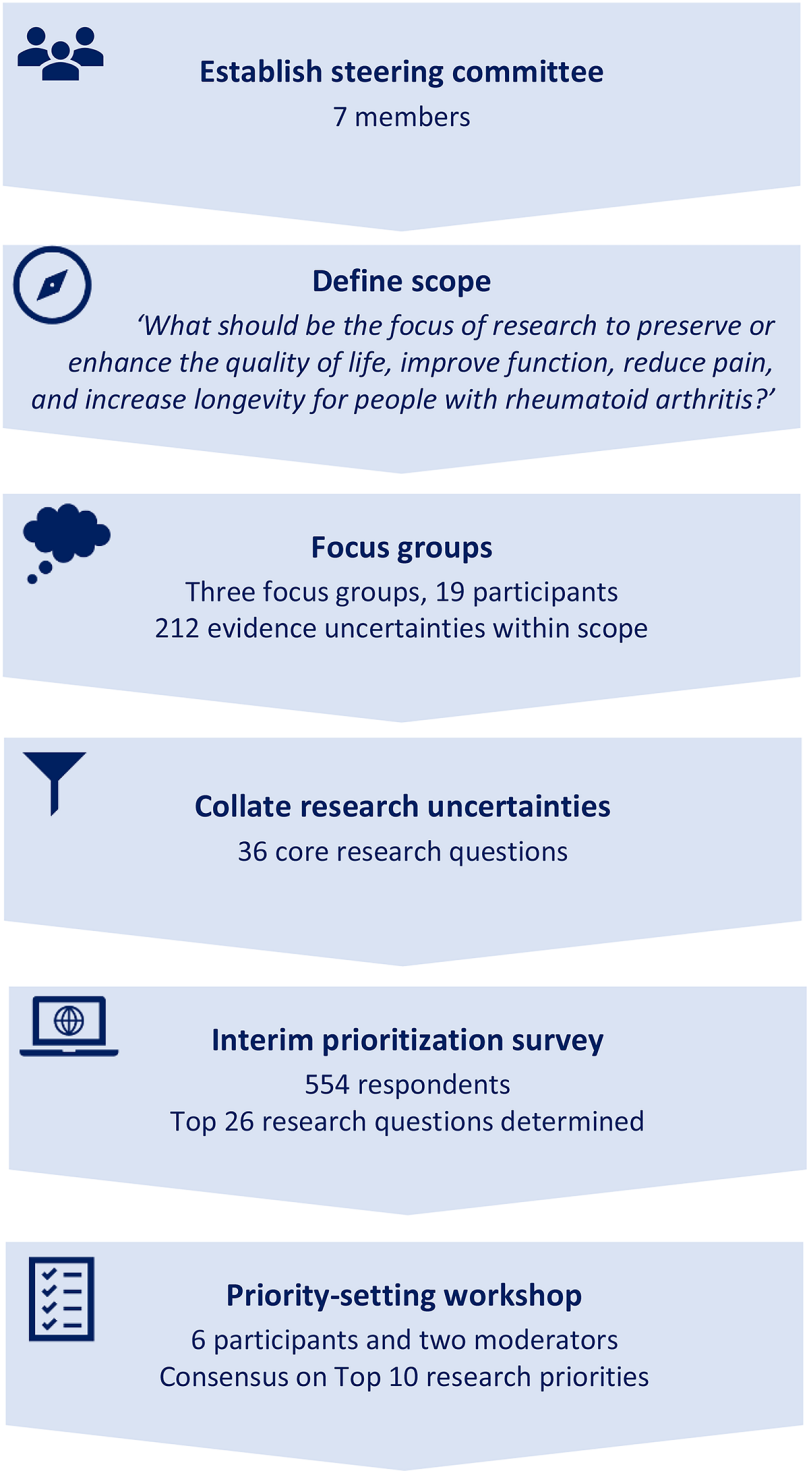
Steps in the James Lind Alliance priority setting partnership for rheumatoid arthritis

The study timeline spanned from September 2024 to January 2025, following the six steps outlined in Fig. 1.

### Identifying and collating evidence uncertainties

The three focus groups consisted of a total of eight people with RA (one male and seven females) in the in-person meetings, seven people with RA (two males and five females) in the digital meetings, and four rheumatologists (three males and one female) in the meeting with healthcare professionals. During these meetings, 58, 90, and 66 evidence uncertainties were identified, respectively. Of these, two were deemed outside the project’s scope. The remaining 212 uncertainties were consolidated into 36 research questions, which were included in an interim prioritization survey with approval from the steering committee. Importantly, none of these research questions were considered sufficiently answered by existing systematic reviews.

### Interim prioritization survey

The online survey attracted 557 respondents. Three respondents did not provide answers to any of the survey items and were consequently excluded from further analysis. This left a final sample of 554 participants, with a mean (SD) age of 58 (13) years (Table 2). Among these respondents, 481 (87%) were female, 449 (81%) identified as people living with RA, and 105 (19%) as healthcare professionals, with some reporting dual roles. A majority of the people living with RA reported a disease duration of more than 10 years.

**Table 2:** Demographic characteristics of respondents from the interim prioritization survey

	All respondents (*n* = 554)	People living with RA	Healthcare professionals (*n* = 105)	Missing, *n* (%)
		(*n* = 449)		
Female, n (%)	481 (87)	399 (89)	82 (78)	
Age, years, mean (SD)	58 (13)	61 (12)	48 (12)	14 (3)
RA disease duration, n (%)				
0–3 months		2 (0.4)		
3–12 months		6 (1.3)		
1–3 years		20 (4.5)		
3–5 years		36 (8.0)		
5–10 years		83 (18.5)		
> 10 years		302 (67.2)		
Type of healthcare professional, n (%)				3 (2.8)
Rheumatologist			47 (46)	
Other type of physician			4 (3.9)	
Physiotherapist			14 (13.7)	
Occupational therapist			21 (20.6)	
Nurse			12 (11.8)	
Social worker			1 (1.0)	
Other (not specified)			3 (2.9)	
Competence in RA, n (%)				3 (2.8)
Very high			24 (23.5)	
High			53 (52.0)	
Moderate			24 (23.5)	
Low			1 (1.0)	
Very low			0 (0)	

Overall mean (SD) rating for the theme categories were: Theme category 3 (*Treatment and side effects*) = 4.74 (0.50); Theme category 1 (*Causation, risk factors, prevention and prognosis*) = 4.64 (0.63); Theme category 2 (*Examination and diagnosis*) = 4.57 (0.68); Theme category 5 (*Societal measures and structure of the healthcare system*) = 4.05 (0.96); and Theme category 4 (*Recognition and attitudes*) = 3.95 (0.97).

Among the top 20 research questions with the highest overall mean scores, two questions tied for 20th place: one from the list adjusted for theme category rating and one from the unadjusted list. To ensure a comprehensive evaluation and capture important topics, we included 21 research questions from both the adjusted and unadjusted lists in the final priority-setting workshop. This allowed us to consider questions that received high ratings without adjustment, even if they scored lower after adjustments. As a result of overlaps between the two lists, a total of 26 unique questions were submitted to the final workshop: 16 questions were taken from both the top 21 list with adjustment and the 21 unadjusted list, while five were unique to the top 21 adjusted list and another five were unique to the top 21 unadjusted. Table 3 displays the overall mean (SD) score for each of these questions without adjustment, as well as separate scores for people living with RA and healthcare professionals. Interim ranks (1–36) for all research questions, shown without and with adjustment for mean theme category score, are also displayed in Table 3. The mean (SD) scores and ranks (1–36) for the 10 questions that were part of the interim survey, but not included in the priority-setting workshop are available in supplementary file 2.

**Table 3:** The 26 highest rated research questions from the interim prioritization survey along with the interim ranking

Research question	Interim mean (SD) score without adjustment 1–5: 1 = Unimportant, 5 = Very important			Interim ranking	
	All respondents	People living with RA	Healthcare professionals	Unadjusted for theme category	Adjusted for theme category
How can we ensure rapid diagnosis of RA?	4.65 (0.70)	4.72 (0.60)	4.34 (0.95)	1	8
How can we prevent RA?	4.63 (0.71)	4.71 (0.61)	4.28 (0.95)	2	2
Which RA treatment works best for whom?	4.58 (0.68)	4.60 (0.67)	4.46 (0.74)	3	1
Why do the symptoms of RA vary and which factors can increase disease activity and symptoms in RA?	4.56 (0.68)	4.62 (0.63)	4.32 (0.81)	4	9
What are the long-term consequences of RA?	4.54 (0.72)	4.63 (0.68)	4.16 (0.76)	5	10
What factors influence the treatment effect of medications in RA?	4.53 (0.68)	4.58 (0.64)	4.32 (0.78)	6	3
What measures can reduce the need for medication in RA?	4.52 (0.77)	4.62 (0.69)	4.11 (0.96)	Joint 7	Joint 4
How to ensure a holistic approach in the treatment of RA?	4.52 (0.79)	4.61 (0.67)	4.13 (1.08)	Joint 7	Joint 4
Can RA be cured?	4.52 (0.88)	4.60 (0.80)	4.16 (1.08)	Joint 7	Joint 4
What are the side effects of RA treatment and how can they be reduced or avoided?	4.51 (0.73)	4.63 (0.60)	4.00 (0.98)	10	7
What is the cause of RA?	4.50 (0.80)	4.56 (0.77)	4.27 (0.89)	11	11
Which factors increase the risk of RA?	4.48 (0.76)	4.54 (0.73)	4.22 (0.86)	12	12
How to increase knowledge among people with RA so that they can make more informed choices in their own lives?	4.45 (0.79)	4.48 (0.78)	4.30 (0.83)	13	23
Is RA a risk factor for other diseases and ailments?	4.42 (0.78)	4.54 (0.68)	3.92 (0.96)	14	14
What types of physical activity and exercise are beneficial for people with RA?	4.38 (0.81)	4.43 (0.78)	4.17 (0.92)	15	13
Will faster assessment and treatment in specialized healthcare improve treatment outcomes for RA?	4.32 (0.93)	4.43 (0.83)	3.85 (1.15)	16	24
Which side effects are perceived as most intrusive for people with RA?	4.31 (0.87)	4.42 (0.81)	3.83 (0.94)	17	15
Does the timing of treatment affect the effect of treatment in RA?	4.23 (0.91)	4.28 (0.89)	4.00 (0.97)	Joint 18	16
How to increase coherence in the Norwegian healthcare system regarding the diagnosis and treatment of people with RA?	4.23 (0.96)	4.37 (0.85)	3.61 (1.14)	Joint 18	25
What treatment do people with RA receive in the Norwegian healthcare system?	4.22 (0.98)	4.37 (0.85)	3.54 (1.17)	Joint 20	Joint 26
How can the Norwegian healthcare system be organized to improve the diagnosis and treatment of RA?	4.22 (0.95)	4.34 (0.87)	3.68 (1.08)	Joint 20	Joint 26
What should be included in a tool for general practitioners for assessing RA?	4.17 (0.98)	4.28 (0.90)	3.71 (1.19)	23	17
Is there a connection between gut health and RA?	4.07 (1.03)	4.16 (1.01)	3.70 (1.02)	Joint 26	18
Is there a connection between hormones and RA?	4.04 (1.00)	4.12 (0.99)	3.67 (0.95)	Joint 28	19
What is the effect of using a tool for general practitioners for assessing RA?	4.04 (1.02)	4.16 (0.96)	3.55 (1.15)	Joint 28	Joint 20
How can we best measure self-reported disease burden in RA?	4.04 (0.93)	4.08 (0.92)	3.88 (0.92)	Joint 28	Joint 20

RA, rheumatoid arthritis

### Priority-setting workshop

The priority-setting workshop was held on January 22nd, 2025 and lasted six hours. The workshop was attended by four people living with RA who had prior experience as patient research partners, two healthcare professionals (one nurse and one physiotherapist), and two moderators (the project leader RMK and deputy project leader ATL). A consensus was reached on the top 10 research priorities for RA as shown in Table 4, and no voting was necessary to achieve this consensus. Additionally, participants agreed that all 26 questions were important, designating the top 10 as ‘gold’ questions and considering the remaining 16 as ‘silver’ questions.

**Table 4:** Top 10 research priorities for RA

Top 10	Research priorities
1	How can we prevent RA?
2	Why do the symptoms of RA vary and what factors can increase disease activity and symptoms in RA?
3	How can we ensure rapid diagnosis of RA?
4	Which RA treatment works best for whom?
5	What are the side effects of RA treatment, and how can they be reduced or avoided?
6	What measures can reduce the need for medication in RA?
7	Is RA a risk factor for other diseases and ailments?
8	How to ensure a holistic approach in RA management?
9	What types of physical activity and exercise are beneficial for people with RA?
10	Is there a connection between gut health and RA?

RA, rheumatoid arthritis

## Discusssion

The field of rheumatology has taken a proactive stance in engaging persons impacted by rheumatic disease in research, yet the participation of people living with rheumatic diseases and healthcare professionals involved in their management in setting research agendas remains limited. To our knowledge, the present study is the first to employ the JLA methodology to identify research priorities in RA. The top 10 research priorities encompass topics related to RA prevention, rapid diagnosis, effective treatment identification, treatment side effects, connections to gut health and comorbidities, and holistic management strategies.

Given the chronic nature of RA, it is unsurprising that questions about disease prevention and treatment efficacy emerged as key research priorities. This focus aligns with priorities identified in psoriatic arthritis and paediatric rheumatology, [15, 16] along with findings from a scoping review on musculoskeletal conditions, which found that nearly half of the research questions centred on treatment [5]. Moreover, the research priorities in psoriatic arthritis and paediatric rheumatology emphasize the identification of factors that predict individual treatment response [15, 16], aligning with one of our top 10 identified research priorities concerning which RA treatments work best for whom. Support for a more personalized treatment approach to RA management is also reflected in EULAR recommendations, which call for the identification of RA disease phenotypes to enable tailored pharmacological interventions [1]. EULAR also advises considering the tapering of disease-modifying anti-rheumatic drugs (DMARDs) in patients who have achieved longstanding remission, while emphasizing the need for additional trials to assess the safety of tapering protocols [1]. This mirrors the clear interest from stakeholders involved in the RA PSP for further research to optimize treatment strategies, including measures that may reduce medication reliance.

Among the top 10 research questions, understanding the variability in RA symptoms reflects an ongoing need to clarify disease aetiology and disease course. The PSP also showed that people living with RA and healthcare professionals recognize the need for more evidence concerning potential treatment side effects. This resonates with EULAR, which stress that although the advent of effective DMARDs have transformed RA management, safety of the various pharmaceutical options at hand needs to be continuously evaluated [1]. 

The non-uniform nature of RA also extends to associated comorbidities and gut health, as reflected in the top 10 research questions. While it is acknowledged that people with RA face an increased risk of several comorbidities, gaps in knowledge persist, particularly regarding how risks in modern-day rheumatology may differ from those of past generations [17, 18]. Additionally, the need to explore potential links to gut health identified in the present PSP was also among the research priorities in psoriatic arthritis and paediatric rheumatology [15, 16] showing a shared interest in understanding the role of the microbiome in rheumatic diseases.

People living with RA have called for a broader treatment approach that extends beyond joint synovitis [19]. Although physical activity and exercise are recommended for persons with RA, systematic reviews emphasize inconsistencies in previous trials and the need for more robust evidence regarding exercise for RA [20] These two issues ranked among the top 10 research questions, emphasizing future research on holistic approaches to RA management and further research into beneficial physical activity and exercise in RA.

A review of PSPs in musculoskeletal disease revealed that questions related to implementation, societal measures, and health service research are often absent from the top 10 research priorities [5]. This trend was also observed in our PSP, where topics concerning recognition and attitudes, societal measures and the structure of the healthcare system that were among the 26 questions in the interim prioritization survey, did not make it into the top 10. However, participants in the priority-setting workshop stressed that the 16 questions that did not make the top 10 list are still important areas for future research, further supported by their high overall ratings in the interim prioritization survey.

Engaging people with lived experiences of a disease and healthcare professionals in identifying research priorities aims to amplify the voices and needs of end-users. We hope the research priorities outlined in this study will guide future endeavours in the field of RA and influence researchers, policymakers, and funding bodies. Beyond disseminating the identified research priorities in RA as an academic publication, we will also share our findings via social media, at relevant congresses, and through patient organizations. Notably, some of the identified research questions align with those proposed in other PSPs and in EULAR recommendations, indicating that the ongoing research agenda in RA is not detached from those impacted by the disease and may reflect an increased emphasis on involving patient research partners in rheumatology [21]. 

Currently, there is limited information on how PSPs have shaped research and funding priorities, and few PSPs having reviewed their outcomes [5, 22]. As a result, the real-world impact of PSPs remains unclear. However, there are novel examples of funding bodies actively looking to support research aligned with priorities derived from JLA PSPs [23]. We welcome future research initiatives to investigate the influence of PSPs on research and funding decisions, for example by tracking the number of grant applications targeting priority topics. Given the rapidly advancing field of rheumatology, identification of research priorities in RA should be updated regularly to reflect the evolving needs of relevant stakeholders, ideally on a 3–5 year cycle [24]. Furthermore, we suggest that future PSPs evaluate the priority setting workshop with specific attention to participants’ perceptions of inclusivity and whether the workshop enabled a balanced exchange of opinions.

### Limitations

A key limitation of the current study is its restriction to Norway. Due to disparities in healthcare systems, access to effective RA treatments, and demographic traits, the research priorities may be less relevant to some other countries. However, we hope that the findings from the Norwegian JLA for RA will inspire other nations to adopt similar approaches that engage people living with rheumatic conditions and clinicians in voicing their desired topics for future research agendas. We aimed to engage various stakeholders in this PSP, but the focus group with healthcare professionals was attended exclusively by rheumatologists. As a result, the research themes voiced in this focus group may not fully represent the topics deemed pertinent among other healthcare professionals involved in RA management. Nevertheless, more than half of the healthcare professionals who responded to the survey came from other professions, ensuring that the rating of research questions extended beyond the input of rheumatologists. Furthermore, the clinicians involved in the priority setting workshop were not rheumatologists, contributing to a broader representation of various healthcare professionals throughout the PSP. 

The interim prioritization survey received more responses from people living with RA than healthcare professionals. Among the people living with RA, most respondents were female, reflecting the higher prevalence of RA in women [25] and many reported a disease duration of 10 years or more. This suggests that the identified research priorities may disproportionately reflect the perspectives of women with longstanding RA, potentially biasing the results. To ensure research priorities reflect the diverse experiences of all those affected by a disease, future PSPs in rheumatology should aim for a more balanced gender representation and actively engage those newly diagnosed. Additionally, the JLA methodology recommends excluding individuals from the pharmaceutical industry and researchers who are not actively engaged in clinical practice. Two of the members of the steering committee (KRN and GB), three of the focus group participants, and two of the participants in the priority-setting workshop were clinicians who hold PhDs and balance research and clinical work. Notably, they were advised to apply the lens of a clinician in all their contribution to the PSP.

In the present study, the research questions generated from focus groups were reviewed against the available evidence by a single reviewer. While this process was thorough, with the steering group consulted whenever uncertainties arose, the trustworthiness of the evidence synthesis could have been enhanced by involving additional reviewers to examine the literature and reach a consensus, similar to the approach used in systematic literature reviews. Another possible limitation is the broad articulation of research questions. While some recommend to use PICO - Population/Problem, Intervention, Comparison, Outcome - to frame research questions, this may result in a narrow scope that is less applicable to qualitative, descriptive and exploratory research designs [24]. Considering the novelty of a PSP in the context of RA, we opted to keep broad questions to portray the current landscape among stakeholders. In line with the JLA guidebook, participants in the focus groups were not required to produce precise research questions; instead, we allowed for questions that captured themes important to people living with RA and clinicians while remaining understandable to non-researchers [9]. We acknowledge that this approach may have resulted in less focused research questions. We recommend that funders and researchers use the identified questions as a starting point and refine them into specific research questions suitable for particular research designs.

Finally, due to the skewed distribution of the respondents’ answers to the interim survey core research questions, it was impossible to rank them using median values. Therefore, we chose to use mean scores. We recognize that using mean summary statistics may be considered inappropriate for ordinal variables, but we determined that this was necessary to enable the ranking process.

## Conclusion

This study, guided by the JLA methodology and using a PSP, identified key research priorities among people living with RA and clinicians involved in its management. Accordingly, future research endeavours in RA should prioritize prevention, rapid diagnosis, effective treatment identification, treatment side effects, connections to gut health and comorbidities, and holistic management approaches.

## Supplementary Information

Below is the link to the electronic supplementary material.

Supplementary Material 1

Supplementary Material 2

## Data Availability

Data supporting the conclusion of this study are available from the corresponding author upon reasonable request and with permission from an ethics committee and Oslo University Hospital (contact through corresponding author).

## Abbreviations

DMARDs: Disease-modifying anti-rheumatic drugs
EULAR: European Alliance of Associations for Rheumatology
JLA: James Lind Alliance
PSP: Priority Setting Partnership
RA: Rheumatoid arthritis
