# Supplementary material for: Mapping the future: identifying research priorities in rheumatoid arthritis with the James Lind Alliance approach
Source: BMC Rheumatol. 2025 Nov 12;9:133. doi: 10.1186/s41927-025-00588-7 (PMC12613741; doi:10.1186/s41927-025-00588-7)
Supplement: Supplementary file 1 — Supplementary Material 1 [file 41927_2025_588_MOESM1_ESM.docx]

## **Supplementary file 1: Outline of focus group meetings**

The focus group meetings are facilitated by the project leader and the deputy project leader, supported by a PowerPoint presentation. The following steps are conducted during the meeting:

## Welcome and introduction

- 1. Introduction of moderators
  2. Ensuring inclusive communication to make sure everyone has a voice

## Purpose of the meeting and focus group

- 1. The importance of including stakeholders—specifically people living with a disease and clinicians involved in its management—in shaping future research agendas
  2. Presentation of the James Line Alliance (JLA) process, with a focus on the steps involved, particularly the focus group

## Project scope introduction

## Overview of the JLA focused on rheumatoid arthritis (RA): *‘What should be the focus of research to preserve or enhance the quality of life, improve function, reduce pain, and increase longevity for people with RA?’*

## Participant introductions

## A brief introductory round for focus group participants to introduce themselves

## Main discussion session.

## Focus group participants are invited to share their suggestions regarding evidence uncertainties related to the question: *‘What should be the focus of research to preserve or enhance the quality of life, improve function, reduce pain, and increase longevity for people with RA?’*

## Moderators record each suggestion as it is presented

## Next steps

- 1. Presentation of what happens next; questions from all focus groups will be collated and checked against project scope. All questions deemed within scope will be checked against existing literature to determine if they have been adequately addressed. Questions that remain unanswered will be included in a survey distributed to people living with RA and healthcare professionals. The final step of the JLA will be a priority-setting workshop before disseminating the findings.

## Closing; acknowledgments and farewells
