# Supplementary material for: Mapping the future: identifying research priorities in rheumatoid arthritis with the James Lind Alliance approach
Source: BMC Rheumatol. 2025 Nov 12;9:133. doi: 10.1186/s41927-025-00588-7 (PMC12613741; doi:10.1186/s41927-025-00588-7)
Supplement: Supplementary file 2 — Supplementary Material 2 [file 41927_2025_588_MOESM2_ESM.docx]

## **Supplementary table: Research questions from the interim prioritization survey that were not included in priority-setting workshop.**

| *Research question* | *Interim mean (SD) score*  *1-5: 1= Unimportant, 5=Very important* | | | *Interim ranking* | |
| --- | --- | --- | --- | --- | --- |
|  | *All respondents* | *Persons living with RA* | *Healthcare professionals* | *Unadjusted for theme category rating* | *Adjusted for theme category rating* |
| How can awareness and understanding of RA be increased within the Norwegian healthcare system? | 4.20 (1.00) | 4.39 (0.85) | 3.41 (1.20) | 22 | 31 |
| How can we ensure the implementation of evidence-based treatments for RA in primary care? | 4.16 (0.94) | 4.24 (0.87) | 3.83 (1.14) | 24 | 29 |
| How can we increase knowledge about RA in school health services and ensure better accommodations for children and young people with RA in schools? | 4.10 (1.05) | 4.25 (0.95) | 3.47 (1.19) | 25 | 30 |
| How can we promote agreement between patients and healthcare professionals regarding appropriate treatment options for RA? | 4.07 (0.97) | 4.17 (0.91) | 3.62 (1.09) | Joint 26 | 32 |
| How does healthcare professionals' attitudes toward RA influence the treatment provided to persons with RA? | 4.04 (1.05) | 4.22 (0.93) | 3.25 (1.18) | Joint 28 | 33 |
| Are there subgroups of RA and should these subgroups be treated differently? | 3.96 (0.98) | 3.90 (0.99) | 4.21 (0.89) | 32 | 22 |
| How can awareness and understanding that men can also have RA be enhanced? | 3.81 (1.17) | 3.03 (1.19) | 3.81 (1.17) | 33 | 35 |
| How can group-based education for individuals with RA be effectively organized, and what impact does it have? | 3.59 (1.04) | 3.61 (1.05) | 3.51 (1.01) | 34 | 28 |
| What is the impact of peer support initiatives for individuals with RA? | 3.43 (1.04) | 3.49 (1.06) | 3.19 (0.96) | 35 | 36 |
| Are there gender differences related to RA? | 3.40 (1.18) | 3.43 (1.19) | 3.26 (1.11) | 36 | 34 |

RA, rheumatoid arthritis
